# Transcription factor Sp1 regulates mitotic chromosome assembly and segregation

**DOI:** 10.1007/s00412-022-00778-z

**Published:** 2022-08-02

**Authors:** Samuel Flashner, Michelle Swift, Aislinn Sowash, Alexander N. Fahmy, Jane Azizkhan-Clifford

**Affiliations:** Department of Biochemistry and Molecular Biology, Drexel University College of Medicine, 245 N 15th Street, MS 497, Philadelphia, PA 19102 USA

**Keywords:** Sp1, Mitosis, Chromosome segregation, Condensin complex I, Transcription factor, Chromosome passenger complex

## Abstract

Aneuploidy is a pervasive feature of cancer cells that results from chromosome missegregation. Several transcription factors have been associated with aneuploidy; however, no studies to date have demonstrated that mammalian transcription factors directly regulate chromosome segregation during mitosis. Here, we demonstrate that the ubiquitously expressed transcription factor specificity protein 1 (Sp1), which we have previously linked to aneuploidy, has a mitosis-specific role regulating chromosome segregation. We find that Sp1 localizes to mitotic centromeres and auxin-induced rapid Sp1 degradation at mitotic onset results in chromosome segregation errors and aberrant mitotic progression. Furthermore, rapid Sp1 degradation results in anomalous mitotic chromosome assembly characterized by loss of condensin complex I localization to mitotic chromosomes and chromosome condensation defects. Consistent with these defects, Sp1 degradation results in reduced chromosome passenger complex activity and histone H3 serine 10 phosphorylation during mitosis, which is essential for condensin complex I recruitment and chromosome condensation. Together, these data provide the first evidence of a mammalian transcription factor acting specifically during mitosis to regulate chromosome segregation.

**Supplementary Information:**

The online version contains supplementary material available at 10.1007/s00412-022-00778-z.

## Introduction

Chromosome segregation errors are detectable in up to 90% of solid tumors (Weaver and Cleveland 2006). Persistent chromosome missegregation is associated with poor patient prognosis, decreased patient survival, intrinsic multidrug resistance, and increased intratumoral heterogeneity (Bakhoum and Landau 2017; A. J. X. Lee et al. 2011; McGranahan et al. 2012). Characterizing the molecular mechanisms governing chromosome segregation is therefore a clinical imperative. Mitotic chromosome assembly is essential for mitotic fidelity, yet the factors mediating this process are incompletely characterized. Transcription factors are the second most abundant class of gene in humans and mediate interphase chromatin dynamics (Venter et al. 2001). Despite the preponderance of circumstantial evidence linking transcription factors to mitotic chromosome assembly, their role in this process is undefined.

Dysregulation of transcription factors is associated with increased chromosome segregation errors in a variety of contexts (Astrinidis et al. 2010; Ishak et al. 2017; H.-S. Lee et al. 2018; Rohrberg et al. 2020; Weiler et al. 2017). However, several barriers have obfuscated the direct role of transcription factors in chromosome segregation. Until recently, transcription factors were believed to be globally evicted from mitotic chromatin and therefore were overlooked during cell division (Djeghloul et al. 2020; Festuccia et al. 2016; Ginno et al. 2018; Kadauke and Blobel 2013; Martínez-Balbás et al. 1995; Teves et al. 2016). Mitotic retention of transcription factors has been implicated in mitotic bookmarking which is required for the maintenance of transcriptional programs into G_1_ (Caravaca et al. 2013; Kadauke et al. 2012; Teves et al. 2016). However, how retention of these factors contributes to mitotic fidelity has not been studied. Additionally, conventional loss-of-function molecular techniques slowly deplete protein levels over several cell divisions, thereby interrupting their transcriptional programs and confounding their mitosis-specific effects. Faithful characterization of mitotic transcription factor function therefore requires more rapid protein depletion immediately prior to mitosis. Recent advancements in degron technology have enabled progress in this field; however, few studies have examined the role of individual transcription factors during mitosis (Natsume and Kanemaki 2017). These studies require an abundance of resources; therefore, which transcription factors are evaluated needs to be judiciously selected. The ideal candidates have been implicated in chromosome segregation through poorly defined mechanisms and localize to mitotic chromatin for poorly defined purpose. Specificity protein 1 (Sp1) is a ubiquitously expressed mammalian transcription factor that protects genomic integrity through diverse mechanisms (Astrinidis et al. 2010; Swift et al. 2021a, b, c; Torabi et al. 2018). RNAi-mediated depletion of Sp1 results in chromosome missegregation (Astrinidis et al. 2010). While recent evidence suggests that Sp1 localizes to mitotic chromatin, its precise role during mitosis is undefined (Ginno et al. 2018; Teves et al. 2016). Sp1 is therefore an ideal candidate for the current study in which we interrogate the mitosis-specific role of the transcription factor Sp1 by rapidly degrading it immediately prior to mitotic entry.

Transcription factors direct the organization and assembly of interphase chromatin; however, their role in these processes during mitosis is undefined (Seungsoo Kim and Shendure 2019). Errorless chromosome segregation requires the proper assembly of mitotic chromosomes by condensin complexes I and II (Hirano 2016). Both condensin complexes localize along the chromosome arms and reorganize chromatin early in mitosis. Condensin complexes I and II have distinct functions and dynamics during mitosis (Hirota et al. 2004). Condensin complex II stably associates with chromosomes during DNA replication and axially compacts chromatin in prophase (Ono et al. 2013; Walther et al. 2018). Condensin complex I dynamically associates with mitotic chromosome arms after nuclear envelope breakdown and drives lateral chromosome compaction during mitosis (Gerlich et al. 2006; Walther et al. 2018). While both condensin complexes contain the chromosomal ATPases SMC2 and SMC4, the complexes are composed of unique subunits: nCAP-D2, nCAP-G, and nCAP-H comprise condensin I, while nCAP-D3, nCAP-G2, and nCAP-H2 comprise condensin II (Hirano, 2012). Preventing the association of either condensin complex I or II results in chromosome segregation errors in tissue culture cells (Booth et al. 2016; Samoshkin et al. 2009). Furthermore, condensin complex function is clinically relevant; defective chromosome condensation is associated with leukemia and other disease states (Molina et al. 2020; Woodward et al. 2016). Ultimately, despite progress elucidating the molecular mechanisms promoting chromosome targeting of condensin complex I and II to chromosomes during mitosis, more work is needed to fully characterize the molecular mechanisms driving mitotic chromosome assembly (Hirano, 2016; Robellet et al. 2017; Paulson et al. 2021).

Aurora B kinase is required for condensin-mediated mitotic chromosome assembly through poorly defined mechanisms (Lipp et al. 2007; Takemoto et al. 2007). Aurora B, Borealin, INCENP, and survivin comprise the chromosomal passenger complex (CPC), which is required for chromosome condensation, correcting microtubule/kinetochore attachments, and cytokinesis (Carmena et al. 2012). CPC activity and function are tightly coupled to its spatiotemporal localization. Early in mitosis, the CPC localizes to chromosome arms in a process required for chromosome condensation. However, little is known about how the CPC is targeted to the chromatin in prophase. Loss of Aurora B early in mitosis results in a variety of deleterious phenotypes that phenocopy chromosome condensation defects (Molina et al. 2020; Samoshkin et al. 2009). More work is needed to fully characterize the Aurora B — condensin signaling axis in chromosome segregation.

In this study, we find that transcription factor Sp1 mediates Aurora B localization to mitotic arms and the recruitment of condensin complex I. Auxin-induced rapid Sp1 degradation immediately prior to mitosis results in mitotic defects and chromosome segregation errors. Taken together, we implicate a ubiquitously expressed transcription factor in a clinically relevant, yet poorly understood phenomenon. These data challenge the current paradigm that transcription factors have no direct role in promoting mitotic fidelity.

## Results

### Sp1 dynamically localizes to mitotic centromeres

**Fig. 1:**
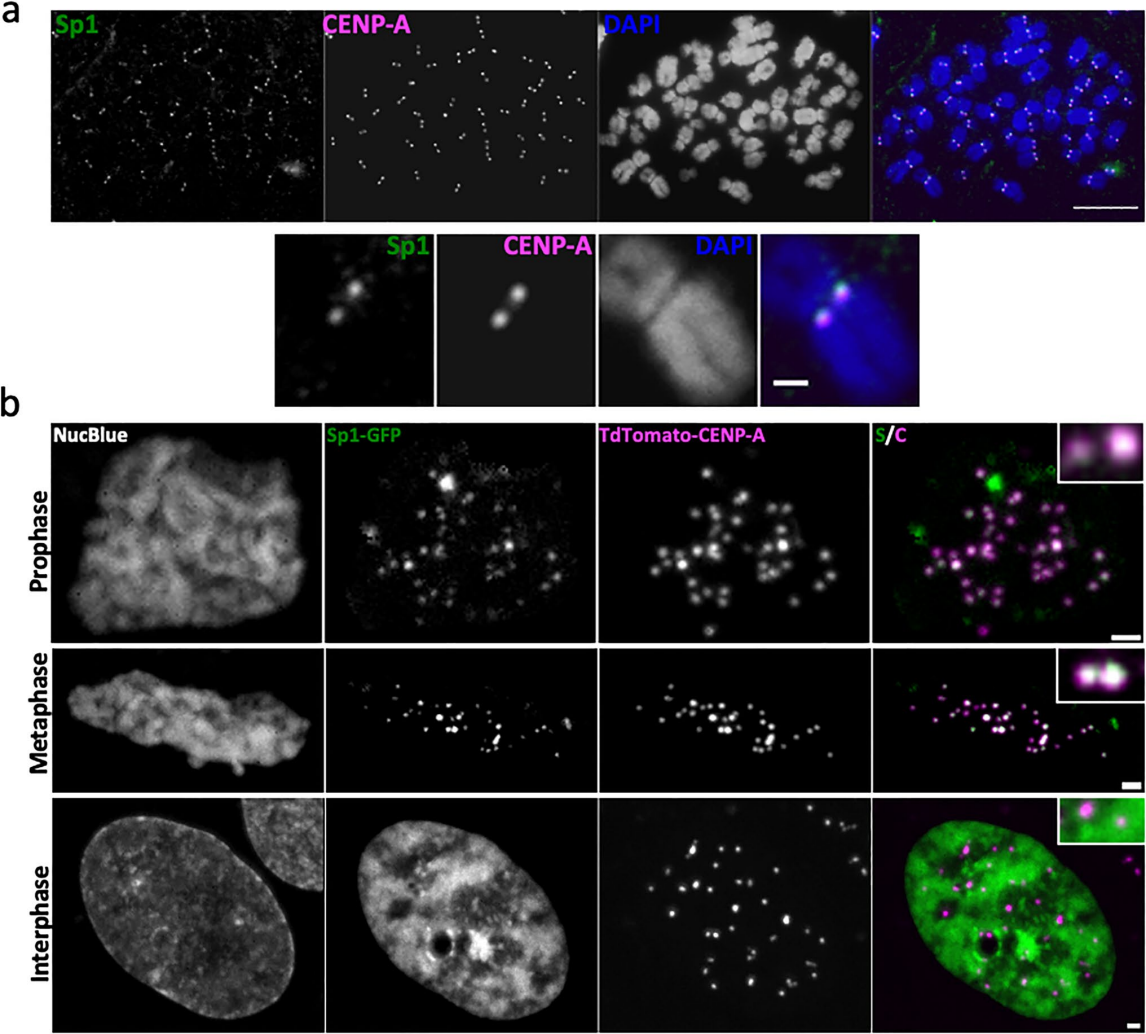
Sp1 localizes to mitotic centromeres. **a** Top: RPE-1 cells were arrested in metaphase, spread onto a glass slide, and stained for Sp1 and CENP-A. Scale bar = 10 µm. Bottom: Representative chromosome. Scale bar = 1 µm. **b** Images taken in live RPE1^TdTomato−CENP−A^ cells transfected with Sp1-GFP. Cell cycle phase was scored based on chromosome conformation. Insets are representative cropped images of Sp1-GFP and TdTomato-CENP-A foci. Scale bar = 2.5 µm

Sp1 was previously shown to localize to mitotic chromatin (Ginno et al. 2018; Teves et al. 2016). While Sp1 is predicted to function as a bookmarker at mitotic chromatin, this hypothesis has not been tested (Ginno et al. 2018; Teves et al. 2016). In order to evaluate this possibility, we first examined Sp1 localization dynamics during mitosis. While previous work demonstrated that Sp1 localizes along entire metaphase chromosome arms (Teves et al. 2016), we found that Sp1 colocalizes with centromeres dynamically during mitosis (Fig. 1). Sp1 colocalizes with CENP-A, a marker of the centromere, in metaphase retinal pigment epithelial (RPE-1) cells (Fig. 1a). We next examined Sp1 localization at different stages of mitosis in live RPE1^TdTomato−CENP−A^ cells transfected with GFP-Sp1 (Fig. 1b). Sp1 co-stains with the centromere marker CENP-A during prophase and metaphase but not in interphase (Fig. 1b), indicating that this dynamic localization occurs specifically in mitotic cells. Finally, we observe no GFP foci in untransfected RPE1^TdTomato−CENP−A^ cells, indicating that the signal present in Fig. 1b is specific to Sp1 (Supplemental Fig. 1). Together, these data indicate that Sp1 dynamically localizes to the centromere during mitotic progression and may therefore have a novel mitosis-specific function.

### Sp1 regulates chromosome segregation during mitosis

**Fig. 2:**
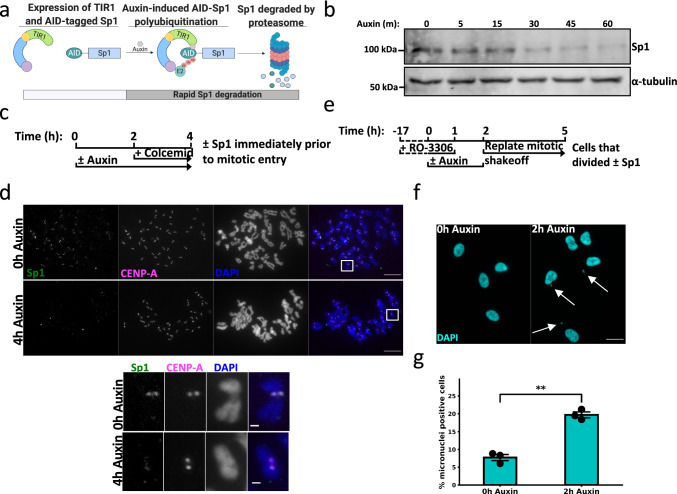
Sp1 regulates chromosome segregation during mitosis. **a** Schematic describing mAID-Sp1 protein degradation. Created with Biorender.com. **b** Immunoblot for the indicated proteins in mAID-Sp1 cells in response to 500 µM auxin. Protein lysates were collected at the indicated time points. **c** Schematic outlining the experimental strategy for (**d**). Upper panel: mAID-Sp1 cells were arrested in metaphase and collected following the described protocol in (Fig. 1c), spread onto a glass slide, and stained for Sp1 and CENP-A. Scale bars = 10 µm. Squares indicate the inset (lower panel). Scale bars = 1 µm. **e** Schematic outlining the experimental strategy for (**f**). **f** Fluorescent detection of DAPI-stained interphase chromosomes following the indicated treatment. **g** The percentage of cells harboring micronuclei (white arrows). Scale bar = 10 µm. Minimum 150 cells counted per treatment. *n* = 3. Black circles represent the mean of each biological replicate. Error bars represent s.e.m., *p* = 0.00056, unpaired *t*-test. All images are representative

We next wanted to evaluate whether Sp1 has a novel mitosis-specific function. Because siRNA-mediated Sp1 depletion results in chromosome segregation errors, we hypothesized that Sp1 functions during mitosis to regulate chromosome segregation. However, characterizing the mitotic function of transcription factors is challenging; RNAi or CRISPR-mediated depletion of transcription factors is too slow to separate their mitosis-specific functions from their interphase transcriptional programs. We overcame this limitation by utilizing an auxin-inducible degron (AID) (Clift et al. 2017; Holland et al. 2012; Nishimura et al. 2009) to rapidly deplete the ubiquitously expressed transcription factor Sp1 in the chromosomally stable cell line RPE-1 (mAID-Sp1) (Fig. 2a) (Clift et al. 2017; Holland et al. 2012; Nishimura et al. 2009). We were able to reduce Sp1 protein levels by treating these cells with auxin for 1 h (Fig. 2b). To determine if we could degrade Sp1 on mitotic chromosomes, we treated mAID-Sp1 cells with auxin for 2 h and then arrested them in metaphase with colcemid for an additional 2 h. Following this treatment, Sp1 was depleted at mitotic centromeres (Fig. 2c and d). This treatment strategy ensures that all visualized AID-Sp1 cells enter mitosis shortly after Sp1 has been degraded (Fig. 2c), isolating the mitosis-specific effects of Sp1.

RNAi-mediated Sp1 depletion results in chromosome-segregation errors (Astrinidis et al. 2010). We therefore hypothesized that Sp1 is functioning at mitotic centromeres to maintain mitotic fidelity. We next evaluated whether Sp1 regulates chromosome segregation during mitosis. To assess chromosome segregation without disrupting the mitotic spindle, we arrested mAID-Sp1 cells at G_2_/M by inhibiting CDK1 with 7.5 µM RO-3306 (CDK1i). We treated arrested cells with auxin for 1 h and then released the cells into mitosis by washing out CDK1i. One hour after release, we isolated mitotic cells by shakeoff and allowed them to enter G_1_. This strategy ensures that we are only evaluating cells that have undergone mitosis shortly after Sp1 was degraded (Fig. 2e). We then quantified the percentage of cells containing micronuclei, a marker of chromosome segregation errors. We determined that rapid Sp1 depletion results in a statistically significant increase in the percentage of micronuclei positive cells, indicating that Sp1 regulates chromosome segregation during mitosis (Fig. 2f and g, Supplemental Fig. 4a). Given our previous findings that long term, RNAi-mediated Sp1 depletion results in chromosome segregation errors concomitant with centrosome amplification, we next assessed whether acute Sp1 depletion results in centrosome amplification specifically during mitosis (Astrinidis et al. 2010). However, we observed no change in centrosome number in cells that had undergone a single cell division immediately following rapid Sp1 degradation (Supplemental Fig. 3), indicating that Sp1 is not regulating chromosome segregation during mitosis by preventing centrosome amplification.

**Fig. 3:**
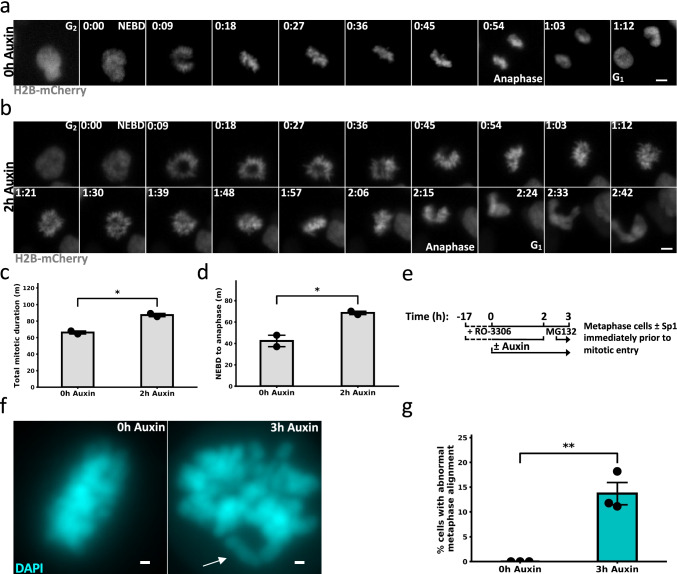
Sp1 regulates mitotic progression. **a** and **b** Live cell imaging of mAID-Sp1; H2B-mCherry cells following the indicated treatments. While images were taken every 3 min, the above image sequence represents images taken every 9 min to best highlight the differences between the treatments. Time = h:min., Scale bar = 5 µm. **c** Time (m) from nuclear envelope breakdown to G_1_. Forty cells counted per treatment. *n* = 2. Black circles represent the mean of each biological replicate. Error bars represent s.e.m., *p* = 0.016, unpaired *t*-test. **d** Time (m) from nuclear envelope breakdown to anaphase. Forty cells counted per treatment. *n* = 2. Black circles represent the mean of each biological replicate. Error bars represent s.e.m., *p* = 0.042, unpaired *t*-test. **e** Schematic outlining the experimental strategy for (**f**). **f** Fluorescent detection of DAPI-stained chromosomes in mAID-Sp1 cells that were arrested in metaphase with MG132. Misaligned (white arrow) chromosomes are completely distinguishable from the metaphase plate. Scale bar = 1 µm. **f** Quantification of (**e**). Minimum 30 cells counted per treatment. *n* = 3. Black circles represent the mean of each biological replicate. Error bars represent s.e.m., *p* = 0.0037, unpaired *t*-test. All images are representative

Together, we show that Sp1 dynamically localizes to mitotic centromeres and regulates chromosome segregation during mitosis.

### Sp1 regulates mitotic progression

To gain a more comprehensive understanding of Sp1’s role during mitosis, we evaluated mitotic progression in live cells. To visualize whole chromosome segregation in the absence of Sp1, we depleted Sp1 in mAID-Sp1 RPE-1 cells that express H2B-mCherry (mAID-Sp1; H2B-mCherry) by treating with auxin for 2 h. We then imaged these cells every 3 min for 4 h (Fig. 3a and b, Supplemental Fig. 4b, and Movies 1 and 2). Rapid Sp1 depletion results in several aberrant mitotic phenotypes. First, Sp1 depletion results in dramatic increases in both total mitotic duration and time from nuclear envelope breakdown (NEBD) to anaphase (Fig. 3c and d). The difference in NEBD to anaphase is similar to the difference in total mitotic duration, indicating that Sp1 is regulating mitotic progression from NEBD to anaphase. Rapid Sp1 depletion appears to result in prolonged monopolar spindle formation (Fig. 3b). Furthermore, careful evaluation of these image sequences revealed that Sp1 fails to properly align along the metaphase plate (Fig. 3b). To quantify this phenotype, we arrested mAID-Sp1 cells at the G_2_/M checkpoint by CDKi, depleted Sp1 with auxin, and released these cells into mitosis and arrested them in metaphase by treating with 10 µm MG132 for 30 min after release (Fig. 3e). This strategy ensured that we evaluated the chromosome alignment specifically in metaphase cells that had progressed through mitosis without Sp1. We then quantified the percentage of these cells harboring rogue chromosomes separate from the primary cluster of chromosomes. We determined that Sp1 regulates metaphase alignment during mitosis (Fig. 3f and g).

### Sp1 regulates mitotic chromosome assembly though condensin complex I localization

**Fig. 4:**
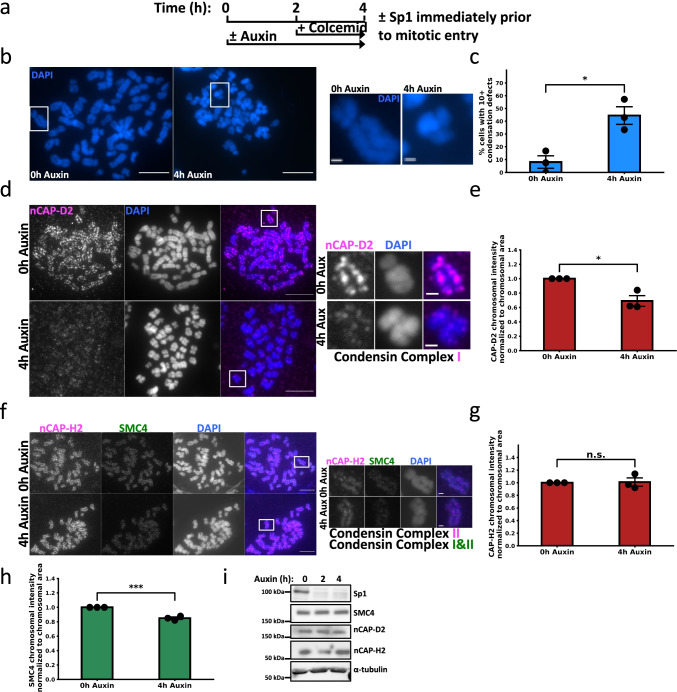
Sp1 regulates mitotic chromosome assembly though condensin complex I localization. **a** Schematic detailing the experimental strategy for panels **b**–**g**. **b** Left, fluorescent detection of DAPI-stained chromosomes in mAID-Sp1 cells following the indicated treatment. Scale bar = 10 µm. Squares indicate the inset (right). Scale bar = 1 µm. **c** Quantification of (**b**). Minimum 28 cells (1288 estimated chromosomes) counted per treatment. *n* = 3. Black circles represent the mean of each biological replicate. Error bars represent s.e.m. *p* = 0.012, unpaired *t*-test. **d** Left, mAID-Sp1 cells were arrested in metaphase and stained for CAPD-2. Scale bars = 10 µm. Squares indicate the inset (right). Scale bar = 1 µm. **e** Quantification of **(d**). Minimum 11 cells (506 estimated chromosomes) counted per treatment. *n* = 3. Black circles represent the mean of each biological replicate. Error bars represent s.e.m., *p* = 0.015, unpaired *t*-test. Left, mAID-Sp1 cells were arrested in metaphase and stained for CAP-H2 and SMC4. Scale bars = 10 µm. Squares indicate the inset (right). Scale bar = 1 µm. **g** Quantification of CAP-H2 intensity in (**f**). Minimum 19 cells (874 estimated chromosomes) counted per treatment. *n* = 3. Black circles represent the mean of each biological replicate. Error bars represent s.e.m., *p* = 0.89, unpaired *t*-test. **h** Quantification of SMC4 intensity in (**f**). Minimum 19 cells (874 estimated chromosomes) counted per treatment. *n* = 3. Black circles represent the mean of each biological replicate. Error bars represent s.e.m., *p* = 0.00098, unpaired *t*-test. **i** Representative immunoblot for the indicated proteins in mAID-Sp1 cells in response to 500 µM auxin. Protein lysates were collected at the indicated time points

We next investigated how Sp1 regulates chromosome segregation during mitosis. Rapid Sp1 depletion results in micronuclei formation, increased mitotic duration, aberrant metaphase alignment, and monopolar spindle formation. These phenotypes are all associated with defective chromosome condensation (Hirota et al. 2004; Martin et al. 2016; Samejima et al. 2018; Samoshkin et al. 2009). We therefore hypothesized that Sp1 regulates chromosome condensation during mitosis. We first evaluated global mitotic chromosome condensation by quantifying the percentage of cells with a general condensation defect. To do so, we categorized sister chromatid pairs without clearly distinguishable p and q arms as abnormally condensed chromosomes. We considered metaphase spreads with greater than 10 abnormally condensed chromosomes to harbor chromosome condensation defects. Following the protocol in Fig. 4a, we found that rapid depletion of Sp1 immediately prior to mitosis results in defective chromosome condensation (Fig. 4b and c, Supplemental Fig. 4c). We next considered how Sp1 is regulating chromosome condensation by evaluating the localization of condensin complexes I and II to metaphase chromosomes. We found that rapid depletion of Sp1 immediately prior to mitosis results in decreased chromosomally associated nCAP-D2, indicating a loss of condensin complex I (Fig. 4d and e). We did not observe a decrease in nCAP-H2 at mitotic chromosomes in these cells, indicating that condensin complex II localizes to mitotic cells independently of Sp1 (Fig. 4f and 4g). We also detected a decrease in chromosomally associated SMC4, a subunit common to both condensin complexes (Fig. 4h). Importantly, Sp1 depletion does not alter condensin complex protein levels, indicating that Sp1 regulates the chromosomal localization of condensin complex I through a non-transcriptional mechanism (Fig. 4i). Together, these results suggest that Sp1 regulates condensin I-mediated mitotic chromosome condensation.

### Sp1 regulates Aurora B kinase activation early in mitosis

**Fig. 5:**
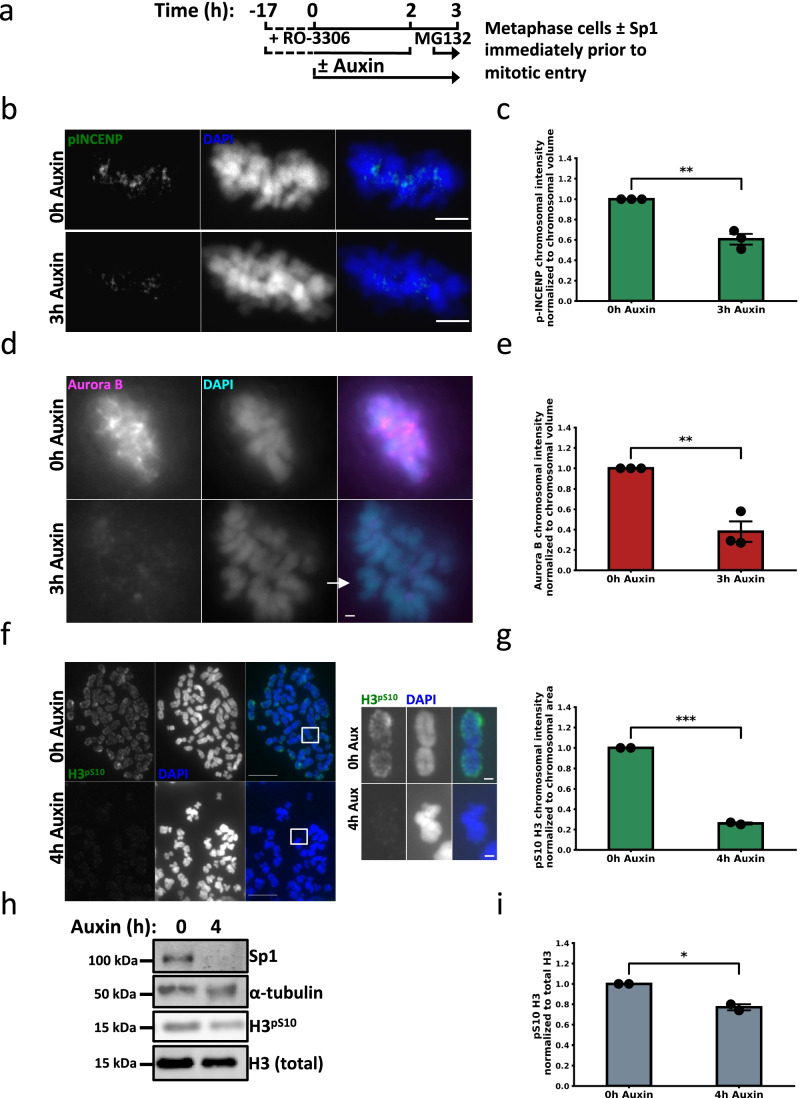
Sp1 regulates Aurora B kinase activation early in mitosis. **a** Schematic detailing the experimental strategy for panels **b**-**e**. **b **mAID-Sp1 cells were arrested in metaphase and stained for p-INCENP. Scale bar = 5 µm. **c** Quantification of (**b**). Minimum 29 cells counted per treatment. *n* = 3. Black circles represent the mean of each biological replicate. Error bars represent s.e.m., *p* = 0.0017, unpaired *t*-test. **d** mAID-Sp1 cells were arrested in metaphase and stained for Aurora B kinase. Scale bar = 1 µm. White arrow indicates a misaligned chromosome. **e** Quantification of (**d**). Minimum 30 cells counted per treatment. *n* = 3. Black circles represent the mean of each biological replicate. Error bars represent s.e.m., *p* = 0.0035, unpaired *t*-test. **f** Left, mAID-Sp1 cells were arrested in metaphase and collected following the described protocol in (Fig. 2c), spread onto a glass slide, and stained for H3^pS10^. Scale bar = 10 µm. Squares indicate the inset (right). Scale bar = 1 µm. **g** Quantification of (**f**). Minimum 19 cells (874 estimated chromosomes) counted per treatment. *n* = 3. Black circles represent the mean of each biological replicate. Error bars represent s.e.m., *p* = 0.00018, unpaired *t*-test. **h** Representative immunoblot for the indicated proteins in mAID-Sp1 cells following treatment with 500 µM auxin. Protein lysates were collected at the indicated time points. **i** Quantification of the densitometry normalized to H3 (total) from (g). *n* = 2. Black circles represent the mean of each biological replicate. Error bars represent s.e.m. *p* = 0.017, unpaired *t*-test

We next evaluated how Sp1 is regulating condensin I-mediated mitotic chromosome assembly. Aurora B kinase is required for the localization of condensin complex I but not complex II to mitotic chromosomes in human cells and in frogs (Lipp et al. 2007; Takemoto et al. 2007). We therefore hypothesized that Sp1 promotes Aurora B kinase activity. Aurora B kinase activity and localization varies through mitotic progression and requires an intact mitotic spindle. We controlled for this variation by first evaluating Aurora B activity in metaphase cells with an intact mitotic spindle using the protocol described in Fig. 5a. We measured Aurora B kinase activity by immunostaining cells with a phosphospecific antibody that recognizes the C-terminal TSS motif of INCENP (p-INCENP), a well-established marker of Aurora B activation (Salimian et al. 2011). We found that rapid Sp1 depletion prior to mitosis results in decreased p-INCENP at metaphase chromosomes (Fig. 5b and c). Aurora B activation is required for its localization to mitotic chromosomes (Salimian et al. 2011). Consistent with these observations, we next found that Sp1 regulates Aurora B kinase localization to metaphase chromosomes (Fig. 5d and e). Importantly, Aurora B is not enriched at the misaligned chromosome (Fig. 5d), indicating that Aurora B is not functioning properly in the absence of Sp1. Additionally, Aurora B is not decreased at the protein level, indicating that Sp1 regulates Aurora B localization to mitotic chromosomes through a non-transcriptional mechanism (Supplemental Fig. 2e). Aurora B kinase activity early in mitosis is responsible for condensin I localization to mitotic chromosomes (Giet and Glover 2001; Lipp et al. 2007; Takemoto et al. 2007). Aurora B phosphorylates histone H3 on serine 10 (H3^pS10^) during late G_2_/early prophase in an event linked to chromosome condensation (Crosio et al. 2002). We therefore next evaluated if Sp1 is required for this phosphorylation. Due to the stability of this mark through metaphase, we rapidly depleted Sp1 immediately prior to mitosis and immunostained metaphase cells for H3^pS10^. We found that rapid depletion of Sp1 results in a dramatic decrease in H3^pS10^, indicating that Sp1 regulates Aurora B activity during the initial phases of mitosis (Fig. 5f and g). We also observed a decrease in H3^pS10^ protein levels, corroborating our IF data (Fig. 5h and i). There is a difference in magnitude between the IF results comparing H3^pS10^ levels in cells following rapid Sp1 depletion (~ 70% reduction in H3^pS10^ protein levels) versus our western blot results (~ 20% in H3^pS10^ protein levels). This discrepancy may the result of technical differences between the two assays or the accidental inclusion of interphase cells in the protein lysate, resulting in dilution of the H3^pS10^ signal and leading to reduced sensitivity in delineating the full difference between the two groups. Despite this discrepancy, both results are statistically significant and indicate a decrease in H3^pS10^ protein levels following rapid Sp1 depletion immediately prior to mitosis. Finally, we evaluated whether Sp1 is preventing the formation of the CPC complex itself by performing a CoIP for Aurora B kinase and western blotting for CPC members following rapid degradation of Sp1 immediately prior to mitotic entry. We found that Sp1 degradation does not alter CPC complex formation, indicating that Sp1 may be responsible for the proper localization of the CPC complex along the chromosome arms early during mitosis and at metaphase centromeres (Supplemental Fig. 4). Together, these results indicate that Sp1 regulates CPC localization early in mitosis.

## Discussion

**Fig. 6:**
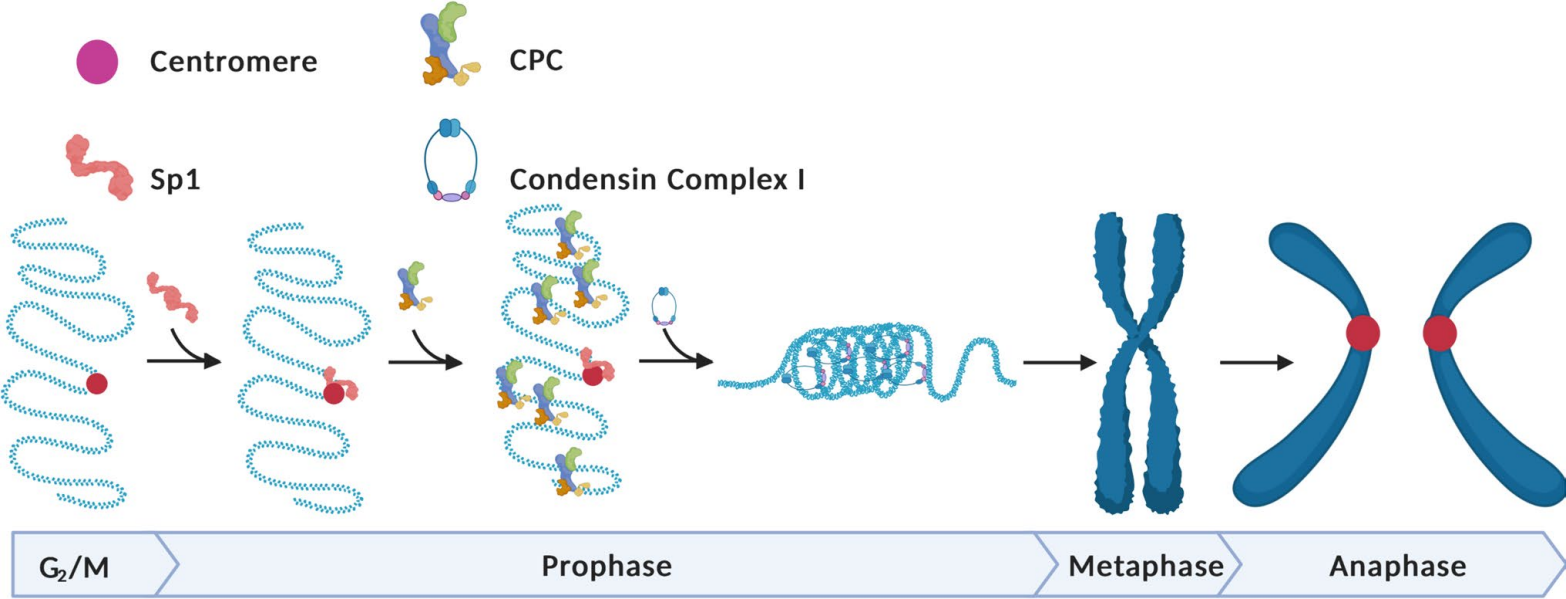
Sp1 regulates mitotic chromosome assembly and segregation during mitosis. Sp1 localizes to mitotic centromeres early in mitosis and regulates Aurora B kinase recruitment, condensin I-mediated chromosome assembly in prophase and ultimately proper segregation of sister chromatids in anaphase. Model created with Biorender.com

Mitotic chromosome segregation and assembly is a complex process with broad pathophysiological implications and therefore constitutes a critical area of molecular research. The role of transcription factors in this process has been largely overlooked. The present study reveals a role for a ubiquitously expressed transcription factor in mitotic chromosome assembly and chromosome segregation. We demonstrate that Sp1 protects genomic integrity during mitosis and promotes mitotic chromosome assembly through Aurora B kinase and condensin complex I recruitment to the chromatin (Fig. 6).

The role of transcription factors in mitotic chromosome segregation and assembly is an emerging area of research. Transcription factors have recently been shown to localize to mitotic chromatin through a highly dynamic process that may be independent of sequence-specific DNA binding (Ginno et al. 2018; Raccaud et al. 2019; Teves et al. 2016). This localization has been implicated in mitotic bookmarking, which is required to maintain transcriptional programs upon entry into G_1_ (Kadauke and Blobel 2013; Liu et al. 2017; Sarnataro et al. 2021; Soares et al. 2021; Teves et al. 2016)_._ Additionally, active transcription of specific genes during mitosis is required for mitotic survival (Dietachmayr et al. 2020). There is now growing support for a more direct role for transcription factors in mitosis-specific processes. In yeast, transcription factors are required for chromosome condensation and mitotic chromosome assembly (Iwasaki et al. 2015; K.-D. Kim et al. 2016; Sutani et al. 2015). In *Xenopus*, the general transcription factor TFIIH is required for condensin I and II deposition on chromatin by dynamically altering the chromatin environment independent of transcription (Haase et al. 2022). However, the role of mammalian transcription factors in these processes is unclear. Here, we implicate a mammalian transcription factor in promoting mitotic chromosome assembly and preserving chromosomal stability.

We have recently characterized cell-cycle regulated functions of the transcription factor Sp1 in preserving genomic integrity. We demonstrated that Sp1 localizes to DNA double stand breaks (DSBs) during G_1_ phase and directly recruits the histone acetyltransferase p300, which generates a permissive chromatin environment for the recruitment of Ku70 and subsequent DSB repair through nonhomologous end joining (Beishline et al. 2012; Swift et al. 2021a, b, c). We have demonstrated that Sp1 is degraded at DSBs at the onset of S-phase through its interaction with RNF4 in a process that is required for homologous recombination (Swift and Azizkhan-Clifford 2022). Together, these data provide precedent for how the cell-cycle dependent chromosomal localization of the transcription factor Sp1 can modulate both the chromatin landscape and the recruitment of specific proteins to preserve genomic integrity though a nontranscriptional mechanism.

We therefore considered whether Sp1 may be performing a similar function during mitosis by evaluating whether Sp1 is mediating the mitotic activity of the chromosome passenger complex (CPC), which is responsible for condensin complex I loading early during mitosis (Lipp et al. 2007; Takemoto et al. 2007). The CPC forms and is recruited to the centromere in late G_2_ before being targeted to prophase chromosome arms to promote mitotic chromosome condensation (Abad et al. 2019; Ainsztein et al. 1998; Seul Kim et al. 2020; Ruppert et al. 2018). Intriguingly, Sp1 depletion immediately prior to mitotic entry does not impair CPC formation (Supplemental Fig. 4). However, rapid Sp1 degradation at the centromere results in loss of CPC activity early during mitosis (Fig. 5f–i), which accounts for the loss of mitotic chromosome assembly (Fig. 4) (Lipp et al. 2007; Takemoto et al. 2007). Consistent with our results, loss of CPC activity partially phenocopies condensin dysfunction, including chromosome segregation errors, aberrant metaphase alignment, and disrupted mitotic chromosome assembly (Figs. 2f and g, 3f and e, and 4) (Adams et al. 2001; Lipp et al. 2007; Martin et al. 2016; Samoshkin et al. 2009). Given that both Sp1 and the CPC are present at the centromere in early mitosis, and loss of Sp1 results in loss of CPC localization along the chromatin arms during prophase, we hypothesize that centromeric Sp1 is mediating centromeric localization of the CPC early during mitosis (Fig. 6). However, how Sp1 may be modulating CPC behavior during this period remains enigmatic and should be the subject of future studies.

Determining how Sp1 is regulating the CPC is challenging. Although the CPC has diverse functions throughout mitotic progression, most studies focus on its role later during mitosis (Carmena et al. 2012). Therefore, little is known about how the CPC is activated and recruited during early mitosis. Emerging evidence suggests that the prophase localization of the CPC requires unique chromatin architecture at both the centromere and along chromosome arms (Abad et al. 2019; Seul Kim et al. 2020). Sp1 is known to interact with a variety of chromatin modifying factors to generate permissive chromatin environments for diverse functions including transcription and DNA repair (Beishline et al. 2013; Beishline and Azizkhan-Clifford 2015; Doetzlhofer et al. 1999; Hung et al. 2006; Kadam et al. 2000). We speculate that Sp1 may be performing a similar function at mitotic centromeres by facilitating the dynamic changes in chromatin required for CPC localization. This characterization is challenging due to the paucity of information about the requirements for CPC localization early during mitosis. Another intriguing possibility is that Sp1 is regulating transcription through the centromere, which is required for Aurora B localization and function in *Xenopus* eggs (Blower, 2016; Grenfell et al. 2016). However, these studies have not evaluated if this transcription is occurring early or later during mitosis. In humans, centromeric transcription is initiated during late mitosis and is therefore not responsible for CPC-induced mitotic chromosome condensation during prophase (Quénet and Dalal 2014). We therefore predict that Sp1 is not regulating CPC-dependent mitotic chromosome assembly by mediating transcription at the centromere. Overall, we implicate the transcription factor Sp1 as a key mediator for CPC localization and function early during mitosis and predict that this function is independent of Sp1’s transcriptional activity (Fig. 6). These observations do not eliminate the possibility that Sp1 may have additional roles during mitosis, including functioning as a mitotic bookmarker or modulating transcription of centromeric DNA later in mitosis. Future studies should address these possibilities.

Our findings may have clinical relevance. Therapeutic strategies targeting chromosome segregation errors represent an emerging area of precision medicine which is hampered by the resource intensive quantification of whole chromosome aneuploidy by karyotyping or DNA sequencing and copy number quantification (Schonhoft et al. 2020). Biomarkers that predict whole chromosome aneuploidy would therefore reduce the severe burden associated with this pervasive phenotype. Future studies will attempt to establish a causative link between loss of Sp1 expression and aneuploidy in human cancers by determining if restoring Sp1 expression levels in primary patient samples can rescue chromosome segregation defects. Strengthening the link between Sp1 and chromosome segregation errors would position Sp1 as a biomarker for chromosome segregation errors in human cancers, which may result in more precise and effective therapeutic interventions.

Mitotic chromosome assembly is also frequently dysregulated in human cancer. In B-cell acute lymphoblastic leukemia, Aurora B-condensin axis impairment is a pathogenic contributor to high-hyperdiploidy, a common and initiating oncogenic event (Molina et al. 2020). Furthermore, loss of condensin complex protein and/or Aurora B kinase levels is associated with chromosome segregation errors and poor patient prognosis. Genes encoding condensin complex proteins are deleted in high percentages of ovarian (97.7%), lung (82.4%), breast (82.3%), and colorectal cancers (54.8%) (Baergen et al. 2019). Additionally, low expression of condensin complex subunits is associated with poor overall survival in colorectal cancer (Baergen et al. 2019). In patients with pyothorax-associated lymphoma, loss-of-function mutations in SMC2 and SMC4 are associated with abnormal mitosis and genomic instability (Ham et al. 2007). Additionally, Aurora B kinase itself is a longstanding target in cancer therapy (Helfrich et al. 2016; Tang et al. 2017; Wilkinson et al. 2007). Loss of Aurora B kinase localization to mitotic centromeres is associated with chromosome segregation errors (Liang et al. 2020). These data highlight the clinical relevance of mitotic chromosome assembly. Extensive characterization of the mechanisms governing this assembly may therefore result in a significant clinical impact in cancer diagnosis and therapy.

We encountered several technical limitations while conducting this study. First, we were unable to detect exactly when Sp1 was evicted from centromeres during mitosis (Fig. 1b). We did not observe any GFP-Sp1 positive anaphase or telophase centromeres but are not confident that this was because Sp1 was evicted from the centromere prior to anaphase entry or if these cells never received the GFP-Sp1 plasmid. However, we did not encounter this issue during interphase as GFP-Sp1 produces an unambiguous signal which does not overlap with CENP-A in any of the observed GFP-Sp1 positive cells. We are therefore confident that Sp1 localizes to the centromere exclusively and dynamically during mitosis.

Additionally, we were unable to generate metaphase spreads from cells previously arrested at G2/M checkpoint. Therefore, we performed all metaphase spread experiments 4 h following the addition of auxin (Figs. 2c and 4a

). Consequently, some of the assayed metaphase cells were in late G2 when we depleted Sp1, raising the possibility that Sp1 may alter chromosome condensation late during G2 either directly or by modulating transcription during this period. We addressed this possibility by quantifying condensin complex protein levels 4 h after Sp1 depletion and found no such change (Fig. 4i). Additionally, Sp1 depletion at the onset of mitosis produces phenotypes consistent with defective chromosome assembly (Figs. 2f and g, 3e–g, and 5a–e). We therefore conclude that Sp1 is modulating chromosome assembly through nontranscriptional mechanisms. These limitations do not alter the central conclusions of this manuscript.

Here, we have demonstrated that the ubiquitously expressed transcription factor Sp1 regulates mitotic chromosome assembly. Loss of Sp1 results in loss of condensin complex I localization to mitotic chromosomes and Aurora B kinase dysfunction early in mitosis. Ultimately, these defects result in aberrant mitotic progression, defective metaphase alignment, and increased chromosome segregation errors. Future experiments should evaluate how Sp1 is mechanistically required for CPC-mediated chromosome assembly, as well as evaluate whether Sp1 has other mitosis-specific roles that promote mitotic fidelity. Ultimately, this work challenges the paradigm that transcription factors are mitotic spectators.

## Materials and methods

### Cell lines

All cells were maintained at 37 °C in a humidified atmosphere with 5% CO_2_. hTERT RPE-1 cells (ATCC) and hTERT RPE-1^TdTomato−CENP−A^ (kind gift of Dr. Iain Cheeseman) were cultured in Dulbecco’s modified of Eagle’s medium/Ham’s F-12 50:50 Mix (Cellgrow) supplemented with 10% fetal bovine serum (FBS; Gemini), and 0.01 mg/ml hygromycin B (Thermo Fisher Scientific). NHDF cells (ATCC) supplemented with 10% FBS. HEK293T cells were maintained in DMEM (Cellgrow) supplemented with 10% heat-inactivated FBS and Pen-Strep. HEK293-GPG were cultured in DMEM (Cellgrow) supplemented with 10% heat-inactivated FBS, Pen-Strep, 1 μg/mL tetracycline, 2 μg/mL puromycin, and 0.3 mg/mL G418. mAID-Sp1 cells were derived by first transducing RPE-1 cells with lentivirus containing sgSp1 and with lentivirus containing mAID-Sp1 downstream of the Sp1 promoter. These cells were then colony selected and screened for mAID-Sp1 expression. mAID-Sp1 expressing cells were then transduced with retrovirus containing osTIR1, challenged with Blasticidin, colony selected, and then screened for rapid depletion of Sp1 in response to auxin treatment. mAID-Sp1 H2B-mCherry cells were derived by transducing mAID-Sp1 cells with lentivirus containing mCherry-H2B.

### Drug treatments

Cells were treated with auxin (Abcam ab146403, dissolved in ddH_2_O), RO-3306 (Selleck 7747, dissolved in DMSO), MG132 (Sigma 10,012,628, dissolved in DMSO, Colcemid (Sigma, dissolved in DMEM), and Biotin (Sigma 29,129, dissolved in DMEM). All negative controls were treated with the equivalent volume of solvent. Note: in Fig. 4b, we treated with MG132 immediately following RO-3306 washout. Because MG132 blocks the auxin-inducible proteasomal degradation of Sp1, degradation of Sp1 was not as effective (evident in Supplemental Fig. 2d). We therefore waited 30 min after RO-3306 washout for subsequent experiments (Figs. 3e and 5d). This waiting period appeared to facilitate Sp1 depletion (Supplemental Fig. 2e).

### Plasmids

Sp1 sgRNA constructs were made using lentiCRISPR v2, a gift from Feng Zhang (Addgene 49535) (Samejima et al. 2018). The Flag-tag in lentiCRISPR v2 with sgRNA constructs was deleted by excising Flag-Cas9 using restriction enzymes Age1 and BamH1. mAID-Sp1 was constructed by cloning the miniAID protein sequence upstream of sgSp1-resistant Sp1 protein sequence into the pLZS-Sp1 vector using Gibson assembly (Sanjana et al. 2014). miniAid was cloned from pcDNA5/FRT EGFP-miniAID, a gift from Andrew Holland (Addgene plasmid # 101714). pLZS was generated by performing Gibson assembly to replace the CMV promoter with the endogenous Sp1 core promoter (− 1612 to + 1) in the pLENTI CMV GFP Zeo vector, a gift from Eric Campeau and Paul Kaufman (Addgene plasmid #17449) (Gibson et al. 2009). pBabe Blast osTIR1-9myc was a gift from Andrew Holland (Addgene plasmid #80073). pLENTI H2B-mCherry was generated by replacing GFP in the pLENTI CMV GFP NEO vector with H2B-mCherry using Gibson assembly. H2B-mCherry was a gift from Robert Benezra (Addgene plasmid #20972) and pLENTI CMV GFP NEO was a gift from Eric Campeau and Paul Kaufman (Addgene plasmid #17447) (Campeau et al. 2009). pCMV3-C-GFPSpark (Sp1-GFP) plasmid was purchased from SinoBiological (HG12024-ACG).

### Indirect immunofluorescence

Cells were arrested in metaphase with 100 ng/mL colcemid (Sigma) for 2 h and collected by mitotic shake off. Pelleted cells were then resuspended in hypotonic solution (10 mM Hepes pH 7.3; 2% FBS; 30 mM glycerol; 1.0 mM CaCl_2_; 0.8 mM MgCl_2_) and incubated at 4 °C for 15 min. Swollen cells were then spun onto a glass microscope slide using a Shandon Cytospin 3 (2000 RPM for 20 min). Cells were then fixed in methanol (− 20 °C for 30 min) and acetone (− 20 °C for 30 s) and allowed to dry at room temperature. Slides were then stored at − 20 °C indefinitely. Alternatively, cells were seeded onto coverslips and grown to ~ 80% confluence. Cells were then washed with PBS, fixed in 4% formaldehyde for 10 min, washed with PBS, permeabilized in PBS + 0.5% Triton X-100, washed in PBST (PBS + 0.1% Tween-20), and blocked overnight in PBST + 3% BSA. Both coverslip-bound cells and metaphase spreads were then incubated (37 °C for 30 min) with the following antibodies: Sp1 (1:200, Santa Cruz sc14027), CENP-A (1:200, Abcam ab13939), nCAP-D2 (1:50, Santa Cruz sc-398850), nCAP-H2 (1:50, Santa Cruz, sc-393333), SMC4 (1:1000, Novus NBP1-86,635), p-INCENP (1:100, kind gift from Dr. Michael Lampson (University of Pennsylvania), see (Salimian et al. 2011) for detailed information regarding antibody generation), Aurora B kinase (1:1000, BD 611082), histone H3^pS10^ (1:100, Cell Signaling #9701 s), and pericentrin (0.1 µg/ml, Abcam ab4448). Cells were then washed with PBST and incubated (37 °C for 30 min) with the following secondary antibodies: α-Rabbit IgG (H + L) Alexa Fluor® 488 conjugate (A21206, 1:1000), α-Mouse IgG (H + L) Alexa Fluor® 594 conjugate (A21203, 1:1000). DNA was stained (37 °C for 5 min) with 1 µg/ml DAPI (Sigma) in PBS. Cells were then washed with PBST, PBS, and H_2_O before being mounted in VectaMount AQ (Vector Laboratories, Inc) and stored at 4 °C indefinitely. Images for Figs. 1d and 2d and f were obtained with the Olympus AX-70 compound microscope and iVision Scientific Image Process software by BioVision Technologies. Images for Fig. 5b were obtained with the Olympus FV3000 confocal microscope. All other images were obtained with the Evos FL compound microscope. All paired images were acquired in parallel using the same microscope settings for channels that are compared in the analysis (e.g., nCAP-D2 in 0 h auxin and 4 h auxin cells).

### Image analysis

All images were processed and analyzed in parallel using ImageJ. Live cell images were corrected for chromatic aberration by first imaging TetraSpeck Fluorescent Microspheres (ThermoFisher T7284) for image calibration. Bead images were captured using the same settings employed for Fig. 1b and used to estimate the degree of x and y offset using beads that represent the relevant spectra for Fig. 1b (505 excitation/515 emission vs 560 excitation/580 emission). Images were then corrected by shifting the green channel 0.09027778 µM in both the x and y position using TransformJ (Meijering et al. 2001). To best highlight the subnuclear localization of Sp1 in live cell images, only the nuclear Sp1 signal was included in Fig. 1b. This was accomplished by multiplying total Sp1 by a nuclear mask generated by thresholding the NucBlue signal so that the only remaining Sp1 signal co-stains with NucBlue. Metaphase spread images were analyzed with the following workflow: define nuclear area by converting thresholded DAPI staining to a mask; quantify that area by adding the total number of pixels in the mask; quantify chromosome-associated antibody of interest (AoI) intensity by multiplying the AoI by the DAPI mask; normalize by dividing chromosome-associated AoI intensity by total nuclear area. Aurora B kinase images were analyzed with the following workflow: define nuclear area by converting thresholded DAPI staining to a mask; quantify that area by adding the total number of pixels in the mask; quantify nuclear Aurora B Kinase intensity by multiplying Aurora B Kinase intensity by the DAPI mask; obtain the volume of these measurements by adding together all nuclear Aurora B Kinase intensities in each z-stack slice. Due to high background, the p-INCENP images were analyzed with the following workflow: define nuclear area by converting thresholded DAPI staining to a mask; quantify that area by adding the total number of pixels in the mask; quantify nuclear p-INCENP by summing the product of p-INCENP intensity and the DAPI mask. Determine non-nuclear p-INCENP intensity (NNI) by subtracting nuclear p-INCENP intensity from total p-INCENP intensity. Determine background intensity/pixel by dividing total NNI by total number of NNI pixels. Determine nuclear background intensity by multiplying background intensity/pixel by nuclear area. Background correct the nuclear p-INCENP by subtracting nuclear background intensity from nuclear p-INCENP intensity. Obtain the volume of these measurements by adding the background corrected nuclear p-INCENP intensity for each slice in the z-stack. Note that rapid SMC2 depletion does not change the total nuclear volume, indicating that normalizing p-INCENP signal to nuclear volume would not be affected by chromosome condensation defects (Samejima et al. 2018).

### Live cell imaging

mAID-Sp1; H2B-mCherry cells were grown in an 8 well Falcon Chambered Cull Culture Slides (Corning 354108) for at least 24 h. Two hours prior to imaging, cells were treated with 500 µm auxin. Images were acquired at 20 × magnification every 3 min using the Evos FL auto microscope with the on-stage incubator maintaining 37 °C. hTERT RPE-1^TdTomato−CENP−A^ cells were grown in an 8 well µ-Slide 8 tissue culture plate (ibidi 80806) for 24 h and transfected with Sp1-GFP plasmid using Lipofectamine LTX with Plus reagent (ThermoFisher A12621) and allowed to recover for 48 h. One drop of NucBlue Live ReadyProbe (ThermoFisher R37605) was added directly to the media to label DNA in live cells, which were subsequently imaged using the Nikon Ti Eclipse, Yokogawa CSU-X1 Spinning Disk Confocal microscope at 60 × magnification with an on-stage incubator maintaining 37 °C.

### Immunoblots

Cells were lysed in 2 × SDS sample buffer (12.5 mM Tris pH 6.8; 20% glycerol; 4% SDS) and boiled. Protein concentration was determined using the Pierce BCA Protein Assay Kit (ThermoFisher 23225). Samples were then supplemented with 5% β-mercaptoethanol, boiled and vortexed. Either 8 or 10 µg of sample was used for subsequent analysis. Proteins were resolved by SDS-PAGE, transferred to polyvinylidene difluoride (PVDF) membrane; the following antibodies were diluted in TBST + 5% BSA: Sp1 Ab581 (1:1000), α-tubulin (1:1000, Cell Signaling #2244), nCAP-D2 (1:1000, Santa Cruz sc-398850), nCAP-H2 (1:1000, Santa Cruz, sc-393333), SMC4 (1:1000, Novus NBP1-86635), Aurora B kinase (1:1000, BD 611082), histone H3^pS10^ (1:1000, Cell Signaling #9701 s), histone H3 (1:1000, Cell Signaling #3638), survivin (1:1000, Santa Cruz sc-17779), Borealin (1:1000, Santa Cruz sc-376635), and INCENP (1:1000, Thermo Fisher 39–2800). Primary antibodies were then recognized by the appropriate secondary antibodies: IRDye 680RD α-Rabbit (1:10,000, Li-COR), IRDye 800CW α-Mouse (1:5000, Li-COR), HRP α-Rabbit (1:2000, Jackson ImmunoResearch Laboratories 711–036-152), and HRP α-Mouse (1:2000, Jackson ImmunoResearch Laboratories 715–056-150). nCAP-D2 and nCAP-H2 were detected with the appropriate HRP-conjugated secondary antibodies; all other primary antibodies were detected with the appropriate IR-conjugated secondary antibodies. IR-conjugated secondary antibodies were detected by the Odyssey imaging system (Li-COR) and HRP-conjugated secondary antibodies were detected by the GeneSys G:Box F3 gel imaging system (Syngene). Protein size was determined by comparing with Precision Plus Protein All Blue Prestained Protein Standard (Bio-Rad 16103773).

### Coimmunoprecipitation

Cells were treated as described in Supplemental Fig. 4a and subject to immunoprecipitation using previously described methods following IP with Aurora B antibody (BD 611,082) or mouse IgG isotype control (Invitrogen 31903) (Swift et al. 2021a, b, c).

### Statistical analysis

All statistical analysis, data handling, and data visualization were performed with Python 3.7.7 (Python Software Foundation) using the following packages: SciPy (1.4.1), Pandas (1.0.3), NumPy (1.18.1), Matplotlib (3.1.3), and iPython (7.13.0). All *p*-values were calculated by the test indicated in the figure legend.

## Supplementary Information

Below is the link to the electronic supplementary material.

ESM 1 Supplementary file1 (Sp1-GFP not detected in untransfected RPE1^TdTomato-CENPA ^cells. Images taken in live RPE1^TdTomato-CENP-A ^cells to demonstrate that GFP signal from Figure 1b is specific for Sp1 localization. Insets are representative cropped images of Sp1-GFP and TdTomato-CENP-A foci. Scale bar = 2.5 μm (PDF 113 KB)

ESM 2 Supplementary file2 (Sp1 does not regulate centrosome number. Rapid Sp1 depletion does not result in centrosome amplification following 1 cell division. **a **Schematic detailing the experimental protocol. **b **Western blot confirming that Sp1 was depleted following addition of Auxin. **c **Representative IF images of pericentrin foci. **d** Quantification of pericentrin foci. No significant difference between the control and experimental group (PDF 95.6 KB)

ESM 3 Supplementary file3 (Sp1 is not required for CPC formation during mitosis. **a **Schematic detailing experimental strategy. Metaphase-arrested cells were collected for CoIP. **b **mAID-Sp1 cells were arrested in metaphase and CPC formation was assessed by immunoprecipitating Aurora B kinase and then immunoblotting for CPC members INCENP, Borealin, and survivin. No difference in CPC complex formation was observed in the absence of Sp1 (PDF 158 KB)

ESM 4 Supplementary file4 (Western blots related to Fig. 1–4. Representative immunoblots from **a **Fig. 2 d, e **b** Fig. 3 a–d **c** Fig. 4 a–g, **d **Fig. 5 a, b and **e **Fig. 3 e, f and Fig. 5 c, d. (PDF 364 KB)

ESM 5 **Movies 1 and 2**. Sp1 regulates mitotic progression. Live cell imaging of mAID-Sp1 H2B-mCherry cells following treatment with ddH2O (Movie 1) or 500 μM Auxin (Movie 2). Images were taken every 3 minutes. Time = h:min. Each video starts nine minutes prior to entry into mitosis. Related to Fig. 3 PDF (AVI 472 kb)

ESM 6 (AVI 172 kb)

## Data Availability

Not applicable.
