# Supplementary figures and images for: Transcription factor Sp1 regulates mitotic chromosome assembly and segregation
Source: Chromosoma. 2022 Aug 2;131(3):175–91. doi: 10.1007/s00412-022-00778-z (PMC9470683; doi:10.1007/s00412-022-00778-z)

NucBlue

GFP-Sp1

TdTomato-CENP-A

S/C

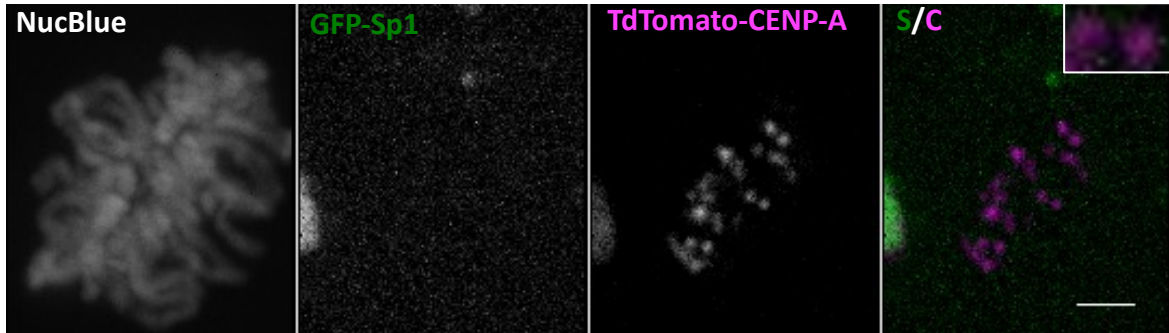

Supplement: Supplementary file 1 — Supplementary file1 (Sp1-GFP not detected in untransfected RPE1TdTomato-CENPA cells. Images taken in live RPE1TdTomato-CENP-A cells to demonstrate that GFP signal from Figure 1b is specific for Sp1 localization. Insets are representative cropped images of Sp1-GFP and TdTomato-CENP-A foci. Scale bar = 2.5 μm (PDF 113 KB) [file 412_2022_778_MOESM1_ESM.pdf]

a

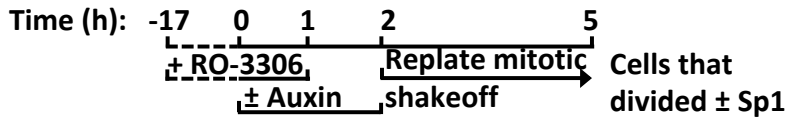

b

1mM Auxin

- +

Sp1

 $\alpha$ -tubulin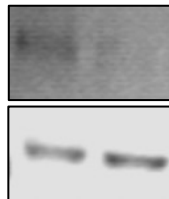

c

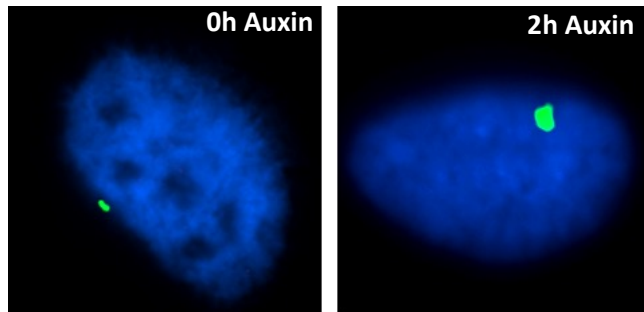

d

Pericentrin foci/cell

1.2  
1.15  
1.1  
1.05  
1  
0.95

1mM Auxin

-

+

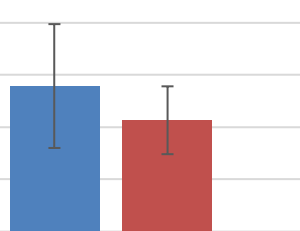

Supplement: Supplementary file 2 — Supplementary file2 (Sp1 does not regulate centrosome number. Rapid Sp1 depletion does not result in centrosome amplification following 1 cell division. a Schematic detailing the experimental protocol. b Western blot confirming that Sp1 was depleted following addition of Auxin. c Representative IF images of pericentrin foci. d Quantification of pericentrin foci. No significant difference between the control and experimental group (PDF 95.6 KB) [file 412_2022_778_MOESM2_ESM.pdf]

**Time (h):** -17      0                  2      3

└───┬──────────────────┐ ┌───┘  
+ RO-3306                  MG132  
└───┬──────────────────┐ ┌───┘  
     ├──────────────────┤ ┌───┘  
     ± Auxin

Metaphase cells ± Sp1 immediately prior to mitotic entry

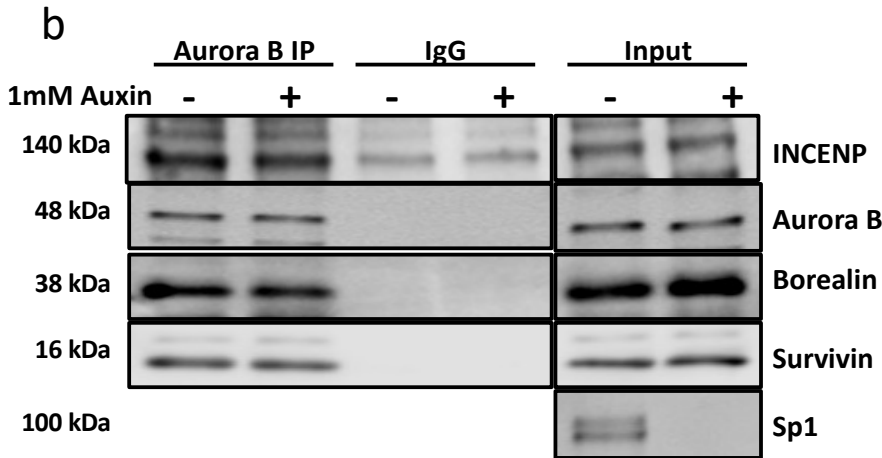

Supplement: Supplementary file 3 — Supplementary file3 (Sp1 is not required for CPC formation during mitosis. a Schematic detailing experimental strategy. Metaphase-arrested cells were collected for CoIP. b mAID-Sp1 cells were arrested in metaphase and CPC formation was assessed by immunoprecipitating Aurora B kinase and then immunoblotting for CPC members INCENP, Borealin, and survivin. No difference in CPC complex formation was observed in the absence of Sp1 (PDF 158 KB) [file 412_2022_778_MOESM3_ESM.pdf]

**a**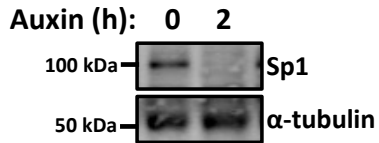**b**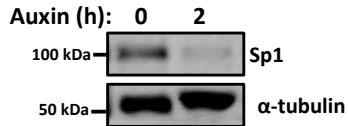**c**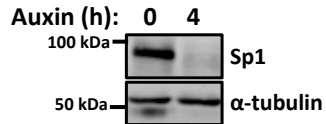**d**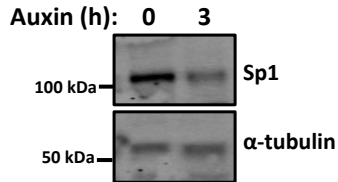**e**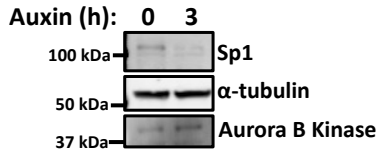

Supplement: Supplementary file 4 — Supplementary file4 (Western blots related to Fig. 1–4. Representative immunoblots from a Fig. 2 d, e b Fig. 3 a–d c Fig. 4 a–g, d Fig. 5 a, b and e Fig. 3 e, f and Fig. 5 c, d. (PDF 364 KB) [file 412_2022_778_MOESM4_ESM.pdf]
